# A qualitative approach to understanding quality symptom management in routine oncology outpatient care: phase 1 of the symptom pathways project

**DOI:** 10.1007/s00520-026-10541-0

**Published:** 2026-04-02

**Authors:** Natasha A Roberts, Diane Maresco-Pennisi, Francesca Boyte, Michael Smith, Helene Jacmon, David Wyld

**Affiliations:** 1 STARS Education and Research Alliance, Metro North Health, Brisbane, Australia; 2 Faculty of Health, Medicine and Behavioural Sciences, The University of Queensland, Herston, Australia; 3 Cancer Care Services, Metro North Health, Brisbane, Australia; 4 Faculty of Nursing, Queensland University of Technology, Brisbane, Australia; 5 Queensland Health, Coopers Plains, Queensland Australia

**Keywords:** Symptoms, Symptom assessment, Oncology, Patients, Carers, Quality of life, Outpatients, Health professionals

## Abstract

**Background:**

The quality of symptom management directly influences the quality of life and survival of patients, a and influences health service outcomes. There is an evidence practice gap between the known benefits of a structured approach to symptom management and how to operationalize high-quality symptom management as a part of routine oncology care.

**Aim:**

To understand practices and processes that influence the quality of symptom management, particularly the characteristics associated with high-quality symptom management.

**Design:**

This work included two qualitative datasets: (i) qualitative focus groups with oncology nurses and (ii) qualitative interviews with specialist health professionals. Positive Deviance Theory informed the methods.

**Setting/participants:**

This study took place in an oncology outpatient department providing care to patients within a major hospital health service in Australia during June 2022 until August 2022. Oncology nurses and specialist health professionals were purposively recruited via email distribution lists and at general staff meetings.

**Results:**

High-quality symptom management practices included early screening and assessment, interprofessional knowledge sharing, referral pathways to specialist teams, and flexible delivery of information to patients and carers.

**Conclusion:**

Quality symptom management may be optimized by drawing on the knowledge of healthcare teams providing symptom management.

**Supplementary Information:**

The online version contains supplementary material available at 10.1007/s00520-026-10541-0.

## Background

Research indicates that symptom management determines the quality of life for those living with cancer [1] and that the prevalence of potentially preventable emergency visits due to cancer-related symptoms is generally high [2]. Unfortunately, moderate to severe symptom burden is overlooked too often and remains a common experience for patients with a range of tumor types, representing a significant unmet need [3, 4]. Common symptoms include anxiety, pain, fever, dyspnea, nausea, diarrhea, fatigue, and constipation. Research aimed at improving how health services deliver symptom management in oncology settings [5] has been called for, as has oncology health delivery that takes a systems-level approach that coordinates the efforts of both patients and healthcare teams [6].

Studies have shown that clear care pathways which proactively respond to and manage patient concerns can result in responsive healthcare delivery in a variety of settings [6–9], for example, pathways providing psycho-oncology care [10]. Conversely, fragmented care can put patients at greater risk of harm, making patients and families feel vulnerable and unsafe [11]. Notably, the cancer care environment is highly complex with multiple elements impacting the delivery of healthcare, making symptom management more challenging, particularly when healthcare teams are required to manage competing priorities in patient care [12].

A project was developed to co-design high-quality symptom management pathways led by a multi-disciplinary team in line with recommendations to take a global approach in oncology care [12]. Quality is defined as “the degree to which health care services increase the likelihood of desired outcomes and are consistent with current professional knowledge” [13–15], and “high quality” is care that is safe, effective, patient-centered, timely, efficient, and equitable [13, 16]. Symptom management is the relief of symptoms from cancer and the side effects caused by any treatments of cancer.

This study is the first phase of a larger multi-phase project designed to improve the quality and reliability of symptom management in oncology care. This project is structured around identifying existing practices that contribute to high-quality symptom management and the co-design structured symptom management pathways to support healthcare teams and patients. The findings from this phase 1 study will inform the subsequent co-design phases, and later phases will involve pilot implementation and evaluation for future scale-up.

The overall aim of this study, as the first phase of the broader project, was to understand current symptom management practices used by oncology healthcare teams. Specifically, the three objectives were to:

i) Determine usual symptom management practices
ii) Define the practices and factors that influence the quality of symptom management
iii) Identify characteristics associated with high-quality symptom management

## Methods

**Fig. 1:**
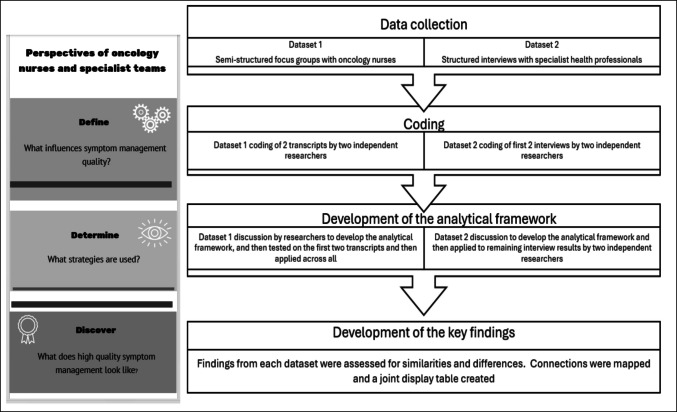
Overview of the study

A qualitative approach was taken using positive deviance theory [17], with analyses informed by a framework method [18]. The methodological approach is presented visually in Fig. 1.

### Setting

This study took place within a Queensland cancer care service at a quaternary referral hospital located within the largest public health service in Australia.

### Participants

A purposive sampling approach was used to recruit participants with direct experience in cancer care delivery. Sampling was stratified by professional role to ensure representation from oncology nurses and specialist health professionals, reflecting their distinct functions within the care pathway.

Recruitment continued until no new findings were identified.

### Guiding theoretical framework

Positive Deviance Theory was used as the theoretical framework for this study [17, 19, 20]. Positive deviance is a strengths-based approach that draws on existing knowledge, behaviors, and successful strategies already present within local healthcare teams. It assumes that healthcare workers may not always recognize the factors that contribute to their own successful practices and that these practices may not yet be formally articulated or embedded in routine care. There are four core stages: (i) define the problem and current practices influencing symptom management; (ii) determine existing strategies; (iii) discover examples of high-quality symptom management; and (iv) develop solutions based on these insights. In this study, Positive Deviance Theory informed the development of the interview and focus group guides by shaping the questions used to elicit examples of effective practices and local solutions. The final “Develop” stage will be reported separately as part of the subsequent co-design process. Although the theoretical framework informed data collection, the analysis itself was conducted inductively using Gale et al.’s framework method [18], allowing key symptom management strategies to emerge from the findings without imposing a priori categories.

### Study procedures

In summary, two datasets were collected. Separate datasets were established for methodological reasons, as the functional nature of the roles in each participant group is distinct. Patients may interact with multiple oncology nurses throughout their cancer journey within a unit that typically operates as a coordinated team. In contrast, specialist health professionals (such as oncologists, allied health clinicians, senior nurse specialists) generally interact with patients and often work independently. To align with these differing practice structures, focus groups were conducted exclusively with oncology nurses, while individual interviews were conducted exclusively with specialist health professionals. There was no overlap between the two participant groups.

Questions for data collection in both datasets included the following:

1. Do you think we have pathways in place for managing symptoms?
2. What formal pathways do we have?
3. What informal pathways are there?
4. What do you think influences the quality of symptom management?
5. What role does each healthcare worker group have?
6. What do you think healthcare workers are doing to ensure the quality of symptom management?
7. What strategies do they use?
8. How do you know that symptom management is of a high quality?
9. How is this achieved?

#### Dataset 1: Oncology nurse focus groups

The focus groups were approximately 45 minutes in length questions in the guide. A semi-structured approach was taken. Each focus group was recorded and transcribed verbatim in a de-identified format.

#### Dataset 2: Interviews with specialist health professionals.

Structured interviews, approximately 30 minutes in length. A structured interview template was used and responses were written down. Participants were given the opportunity to consider and review the written responses and provide further details as required.

### Data analyses

A female clinical research nurse (FB) with experience in qualitative research support roles conducted the focus groups and interviews, supported by an external facilitator clinician researcher with a PhD and 20 years of qualitative research experience (NR). Both were known to healthcare teams. Memos were collected during all meetings for reflexive monitoring and for rigor during reporting by FB and NR. The process for analysis was inductive in nature [21, 22] and informed by the steps outlined in the framework method [18]. Transcripts from dataset 1 were initially coded after two focus groups by both FB and NR independently. An analytical framework was then developed during a meeting with FB and NR and then applied to the first two transcripts again by FB. FB and NR met again and then determined after discussion that it could be applied to the remaining transcripts, with at least two researchers working independently on each transcript.

Data from dataset 2 were initially coded independently by FB and NR from the first two interviews to develop an analytical framework, which was then applied to the remaining interviews. A research assistant (KC) with 2 years qualitative experience and NR conducted an independent application of the analytical framework on all transcripts.

When researchers came together at each step to discuss their findings, disagreements were addressed until consensus was reached. Reflexive memos by FB and NR were discussed with all team members at the step of charting the data into the framework matrix. Dataset 1 and dataset 2 were analyzed separately until stage 6 of the framework method approach (interpreting the findings). These findings were then explored together to interpret the data, drawing upon memos/diarized notes and documented impressions. Similarities and differences were noted; connections were mapped into tables [23].

### Reporting

Reporting of qualitative work used the COnsolidated criteria for REporting Qualitative research (COREQ) [24].

### Ethical considerations

This study received low-risk ethical approval (LNR/2019/QRBW/60332) by the Royal Brisbane and Women’s Hospital Human Research Ethics Committee (EC00172). The research was performed in accordance with the Declaration of Helsinki. One-page information sheets explaining the nature of the project, voluntary participation, and data management were distributed to all staff by email and presented on noticeboards across staff areas. Hard copy information sheets were also provided to healthcare teams and consumer representatives prior to arranging a suitable time for participation in qualitative data collection. The information sheet was re-presented at each data collection so consent could be confirmed. All data were de-identified and stored on password-protected servers in a non-identifiable format. A standard operating procedure for withdrawal from study participation was in place, if required.

## Results

The overall project took place from March 2022 until March 2023. The work in phase 1 presented in this paper took place from June 2022 until August 2022.

### Findings from dataset 1: focus groups to capture the perspectives of oncology nurses

In total, five focus groups (*n* = 5) were conducted involving 27 nurses who were providing care to patients having systemic cancer treatment. All participants were female with an age range of 27 to 48 years. Twenty-one participants had 5–10 years of clinical experience, five had 10–15 years of experience, and one had 20 or more years of experience. The charted data from analyses for dataset 1 is presented in Supplementary File 1.

Participants from dataset 1 reported that they spend time with many patients and their carers during active treatment. Prior to treatment administration, they usually conduct comprehensive symptom assessments using the Common Terminology Criteria for Adverse Events (CTCAE) [25], and will often provide supportive care, largely in the form of education, during treatment administration. If a symptom is assessed to be grade 2 or above, a referral will be initiated.

While barriers to symptom management were identified, enablers relating to the work of members from the healthcare team featured strongly in keeping with the strengths-based approach of positive deviance theory. Nurses also reported that symptom management was largely clinician dependent (including all health professional groups) amid multiple cognitive demands placed on healthcare teams in a health system that is under pressure. “Time, like feeling pressured” (Participant 4, Focus group 4). This was not viewed as a good thing, as it meant that there was a risk that patients can “fall through the gaps” (Participant 2, Focus group 1). The process for referrals was reported to be complex, depending on who it is going to, and complexity or a lack of clarity can influence delivery of symptom management. Traits associated with high-quality symptom management included providing support for patients and carers, identification of symptoms, sharing of information across healthcare teams, bringing in experts as needed, and being responsive (as opposed to being process driven).

### Findings from dataset 2: interviews to capture the perspectives of specialist health professionals

In total, nine interviews were conducted with health team members providing emergent care to patients and carers. Two participants were male and seven were female. The age range was from 32 to 55 years. All participants had 15 + years clinical experience. These health professionals included cancer care coordinators/case managers (*n* = 5) or specialist senior nurses (*n *= 2) who specialized in a specific cancer tumor group, or senior oncology medical officers in training (*n* = 2). Interviews were 15 to 30 min in duration. No participants declined to participate after initial contact or withdrew from the study. Data charted into the framework matrix are presented in Supplementary File 2.

High-quality symptom management was usually delivered by those that had a mastery of symptom management, and worked to ensure that “all is in hand” (Participant 7), even when there are competing pressures to manage. Participants also unanimously agreed that symptom management was largely person dependent and that high-quality symptom management directly influences the outcomes of those in their care. Participants expressed that this responsibility was taken seriously.

Mapping of findings across dataset 1 and dataset 2 identified that symptom management requires assessment as a first step. Communication about the findings of this assessment is often shared with the patient and sometimes the carer, but for high-quality symptom management, this should be repeated on more than one occasion and tailored to the individual health literacy of a patient and/or carer. Interventions to address an identified symptom can vary according to what is identified and the severity. This can be determined by the impact on the patient, their carer, and their ongoing treatment. As needed, timely referrals and communication escalating to specialist services are essential. How these steps seemed to be applied varied and depended on the practice of individual clinicians, with no consistency apparent between and within professional groups.

From the two datasets, strategies that supported high-quality symptom management fell into four key areas:

1. Early screening and assessment in the treatment pathway. Participants spoke about the pathways that patients followed when attending for treatment in the outpatient clinic. Making early screening and assessment available to all health professionals would enable consistent and reliable assessment at every point of interaction along the patient pathway.
2. Multi-modal patient and carer information resources. These resources could be tailored to informational needs and could include verbal, written, online, and co-created information, or any combination of these. Consistency between health professionals providing information was deemed useful.
3. Interdisciplinary knowledge sharing. This included sharing of knowledge specific to patients, such as through a multi-disciplinary team (MDT) meeting approach or morning huddle. Knowledge sharing also included understanding each other’s scope of practice, and evidence-based practice.
4. Formalized referral pathways. These referral processes needed to be accessible to all members of the healthcare team, with feedback mechanisms to the patient’s medical record that a referral had been submitted, and when appropriate, what actions took place.

Table 1 presents a joint display [23] from the findings of dataset 1 and dataset 2 that have informed the findings of the study.

**Table 1:** Joint display of key findings from dataset 1 and dataset 2

High quality symptom management	Dataset 1	Dataset 2	Exemplary quote
Early screening and assessment in the treatment pathway	*Identifying symptoms* Examples: -Early referrals for “at risk” patients -Acting when things “don’t look right” -How assessment questions are constructed -Acting early	-Regular assessments to catch symptoms early -Identifying symptoms and grading symptoms -Dose modifications and other strategies that tailor treatment	“Patients being concerned with the busy rush of the doctor’s clinic and not wanting to divulge the concerns they have with symptoms” Participant 3, Focus group 1
Multi-modal patient and carer information resources	*Supporting patients and carers* Examples: -Nurses advocating for patients -Supporting carer engagement -Checking on shared meanings -Feeling safe *Being responsive* Examples: -Escalating -Listening -Counselling -Tailoring	-Consistency of information provided to patients -Health literacy and other patient related factors -Providing verbal discussion/counselling/education -Time set aside for in-depth discussions -Quick and easy tools that are consistent between healthcare teams and patient resources	“Knowing what to look for. So, education is very important because they know how to look after themselves and what to look for, the symptoms they maybe experience” Participant 2, Focus group 3
Interdisciplinary knowledge sharing	*Sharing information across healthcare teams* Examples: -Documentation processes -Notifying when symptoms are resolved -Trusting each other	-Staff training/expertise -A good multi-disciplinary team where members trust one another -Medical availability to resolve symptoms of concern -Communication tools for the multi-disciplinary team	“I will go and discuss things sometimes with other professionals to understand specific things that patients will tell you” Participant 1, Focus group 3
Formalized referral pathways	*Bringing in the experts* Examples -Access to the multi-disciplinary team -A method to track referrals -Multi-disciplinary approach -Knowing who to send the referral to	-Streamlined pathways for escalation -Structures to support patients between visits -Patient reassurance, normalizing symptoms and having a clear plan	“…breaking down complexity…so you can respond effectively” (Participant 1, Focus group 2)

High-quality symptom management appears to result from both precision in care delivery and the expertise of health professionals involved. High-quality symptom management must take place in partnership with patients and carers, accommodating their specific needs. Patient-focused care delivery, not systems-focused delivery, featured strongly, but was acknowledged to be challenging to deliver consistently currently. In response, clinicians would work to compensate for system limitations, which resulted in symptom management that was clinician dependent.

## Discussion

This paper reports on the first phase of a larger co-design project which aims to optimize high-quality symptom management in an oncology outpatients’ department. Positive Deviance Theory was used to guide the methodological approach. Findings identified that exceptional care includes timely screening and assessment, tailored information resources for patients and carers, interprofessional care, and access to specialist care. Findings across both studies supported an approach to symptom management that is proactive, rather than reactive, in nature.

This work brings new knowledge and a guiding structure for understanding how health professionals provide symptom management in cancer care, particularly in relation to taking a “big picture” approach to symptom management. The importance of “meeting patients and their carers where they are” with tailored education [26] has been previously shown to lead to better patient outcomes and satisfaction [27]. The findings in our study also emphasize the role of the broader multidisciplinary team, which also aligns with work already done by palliative care teams in hospices, who identify that symptom management requires four phases: engagement, decision-making, partnership, and delivery of an agreed plan into action [28]. It also has some synergies with the work of Kelly et al. [29] who did an extensive systematic review and meta-analysis of nurse-led interventions for cancer symptom management, which included studies reported until 2017. Their work identified the potential to improve symptom outcomes, though many interventions reported were poorly defined [29].

Early assessment was a key finding in our study in line with other evidence, for example, work using ePROMs to screen symptoms [1, 12, 13, 30–32]. The need for formalized referral pathways was identified in our work. Despite this appearing intuitive, we were unable to identify literature that explicitly describes this need. The requirement for structured referral pathways, however, does align with other evidence-based interventions such as cancer-specific follow-up and care coordination and structured case management [6, 33]. Participants in our study reported that the complex oncology setting naturally inhibits streamlined access to specialist care, making the need for simple processes vital.

While there are signals in the broader literature that systematic symptom assessment can activate the wider healthcare team [8, 13], symptom assessment without healthcare team support can impair quality of life [34]. It appears that symptom assessment is most effective when supported by intrinsic clinical expertise and mutual trust in interprofessional relationships [18]. Our study has identified that the quality of symptom management is currently dependent on individual clinicians. Research indicates that health delivery that is clinician dependent can result in ad hoc, missed and duplicated care [35]. Optimal cancer care requires systemic structures, and [6] a clear evidence base on how these can integrate with day-to-day cancer care delivery is still forthcoming.

Incorporating a theoretical approach can potentially identify underlying processes and mechanisms for high-quality symptom management that bring together a multi-disciplinary team. The use of the Positive Deviance Theory not only provided structure and rigor to the methods in this work, but, importantly, gave attention to the strengths already in place within the health setting. This approach elevates this work above conventional qualitative methods as it links practices to day-to-day clinical care [25]. High levels of participation by healthcare teams and support by healthcare executives suggest strong engagement, and likely contributed to the feasibility of the project as a whole.

However, this research has limitations that warrant careful consideration. Work was done in a single site. The decision to take a strong theoretical approach supports the internal coherence of the work, supporting the transferability of findings [36]. This work responds to calls for improving healthcare systems to support connected services for managing symptoms [5, 6]. Future research could build upon these findings across multiple centers to establish a robust evidence base which addresses individual and health service outcomes.

## Supplementary Information

Below is the link to the electronic supplementary material.

ESM 1 (DOCX 423 KB)

ESM 2 (DOCX 20.1 KB)

## Data Availability

Data will be made available upon reasonable request.
