# Supplementary material for: A qualitative approach to understanding quality symptom management in routine oncology outpatient care: phase 1 of the symptom pathways project
Source: Support Care Cancer. 2026 Apr 2;34(4):402. doi: 10.1007/s00520-026-10541-0 (PMC13046616; doi:10.1007/s00520-026-10541-0)
Supplement: Supplementary file 1 — (DOCX 423 KB) [file 520_2026_10541_MOESM1_ESM.docx]

Supplementary File 1. Charted data presenting the characteristics, traits and exemplary quotes from qualitative focus groups with oncology nurses charted into the framework matrix.

Characteristics	Traits associated with the theme to support high quality symptom management	Exemplary quote
Supporting patients and carers	Nurses advocating for patients<br>Education for nurses on what to look for<br>Education for patients on how to look after themselves<br>Education for patients on what to expect<br>Supporting carer engagement<br>Education for carers on what resources are available for them<br>Understanding priorities for compliance<br>Tailoring to accommodate carer needs/not having a carer<br>Symptom diaries so patients and carers can self-monitor<br>Responding to what patients and carers tell you<br>Interpreters/resources in the primary language<br>Checking on shared meanings<br>Understanding that some patients pretend they do not have symptoms<br>Patients feeling safe<br>Understanding that not all patients want to be actively involved	*"Knowing what to look for. So, education is very important because they know how to look after themselves and what to look for, the symptoms they maybe experience"*<br>Participant 2, Focus group 3<br><br>*"Control of their own body"*<br>Participant 2, Focus group 2
Identifying symptoms	Access to the multi-disciplinary team<br>Detect changes<br>Gathering more information during ad hoc conversations<br>In depth conversations<br>Specialist skills<br>PROMs fit for purpose	*"I will go and discuss things sometimes with other professionals to understand specific things that patients will tell you"*<br>Participant 1, Focus group 3

	Knowing what to look for<br>Early assessment<br>Educated nursing staff<br>Safety net – we all miss things<br>Patients feeling safe to report symptoms<br>Being thorough<br>Consistency<br>Taking action when *"they don't look right"*<br>How assessment questions are constructed	*""Oh, I've got a bit of pain, but I'm okay. I don't need anything" or maybe because they're not comfortable talking about it"*<br>Participant 4, Focus group 1
Sharing information across healthcare teams	Documentation tools fit for purpose<br>Checklists with open text options<br>Sharing knowledge<br>Documentation<br>Multi-disciplinary discussion<br>Ensuring the multi-disciplinary team can know what is going on, not person dependent<br>Every health care provider who sees a patient, documents that encounter in the medical record<br>Understanding how to escalate a concern<br>Appropriate communication<br>Notifying when symptoms are resolved<br>Trusting each other	*"One of the main things we do is contact the specific expert relating to that symptom in one way or another"*<br>Participant 1, Focus group 4<br><br>*"Patients being concerned with the busy rush of the doctors clinic and not wanting to divulge the concerns they have with symptoms"*<br>Participant 3, Focus group 1
Bringing in the experts	Knowing the right person to send a referral to<br>A designated specialist/expert for each symptom type<br>Multi-disciplinary approach<br>Early referrals for at-risk patients<br>A method to track referrals<br>Making sure additional support is in place early	*"Our dietitians are very visible, we know they are around and so we can easily flag someone we are worried about"*

	A referral platform<br>Allied Health, Specialist Nurses, Medical Specialists<br>Multi-disciplinary team is visible	Participant 1, Focus group 5
Being responsive	Acting early<br>Being resource sensitive (i.e., close management of time, money and staffing)<br>Iteratively finding new ways to improve the health system<br>Escalating<br>Listening<br>Counselling patients and peers<br>Tailoring care	*"…breaking down complexity…so you can respond effectively"*<br>(Participant 1, Focus group 2)
