# Supplementary material for: A qualitative approach to understanding quality symptom management in routine oncology outpatient care: phase 1 of the symptom pathways project
Source: Support Care Cancer. 2026 Apr 2;34(4):402. doi: 10.1007/s00520-026-10541-0 (PMC13046616; doi:10.1007/s00520-026-10541-0)
Supplement: Supplementary file 2 — (DOCX 20.1 KB) [file 520_2026_10541_MOESM2_ESM.docx]

Supplementary File 2. Charted data from findings of interviews with specialist health professionals

| **Symptom management quality:**   1. Patient information 2. Patient related determinants 3. Staff expertise 4. Multi-disciplinary approach | **Exemplary quotes**  *“consistency of information provided to patients”*  *“health literacy”*  *“other patient related factors”*  *“staff training”*  *”expertise”*  *“good multi-disciplinary team”*  *“members trust one another”*  *“medical availability to resolve symptoms of concern”* |
| --- | --- |
| **Roles in symptom management quality**   1. Nurses: Engagement and care 2. Medical: Treatment 3. Allied Health: Symptom control | **Exemplary quotes**  Nurses:  *“early assessment”*  *“building rapport”*  *“delivering interventions”*  *“follow-up”*  *“glue”*  *“patient advocates”*  Medical Officers:  *“diagnosis”*  *“prescribing interventions”*  *“tailoring of dose”*  *“referrals”*  Allied Health:  *“specialised interventions”*  *“information”*  *“reduction of symptom severity”* |
| **Health systems influences:**   1. Assessment structures 2. Referral and escalation mechanisms 3. Interdisciplinary sharing 4. Streamlined systems | **Exemplary quotes**  *“streamlined…escalation”*  *“assessments to catch symptoms early”*  *“time for in-depth discussions”*  *“quick and easy tools”*  *“consistency between healthcare teams”*  *“multi-disciplinary communication”* |
| **Symptom management strategies:**   1. Patient information 2. Assessment 3. Responsive systems 4. Communication | **Exemplary quotes**  *“verbal discussion”*  *“counselling”*  *“education”*  *“identifying symptoms”*  *“grading symptoms”*  *“dose modifications”*  *“other strategies that [tailor] treatment”*  *“structures to support patients between visits”*  *“patient reassurance”*  *“normalizing symptoms”*  *“having a clear plan”* |
